# View Point: Semaphorin-3E: An Emerging Modulator of Natural Killer Cell Functions?

**DOI:** 10.3390/ijms18112337

**Published:** 2017-11-05

**Authors:** Abdulaziz Alamri, Abdelilah Soussi Gounni, Sam K. P. Kung

**Affiliations:** Department of Immunology, Max Rady College of Medicine, Rady Faculty of Health Sciences, University of Manitoba, Winnipeg, MB R3E 0T5, Canada; alamria3@myumanitoba.ca (A.A.); Abdel.Gounni@umanitoba.ca (A.S.G.)

**Keywords:** semaphorins, semaphorin-3E, plexins, nervous system, cardiovascular, allergic asthma, cancer, natural killer cells

## Abstract

Semaphorin-3E (Sema-3E) is a member of a large family of proteins originally identified as axon guidance cues in neural development. It is expressed in different cell types, such as immune cells, cancer cells, neural cells, and epithelial cells. Subsequently, dys-regulation of Sema-3E expression has been reported in various biological processes that range from cancers to autoimmune and allergic diseases. Recent work in our laboratories revealed a critical immunoregulatory role of Sema-3E in experimental allergic asthma. We further speculate possible immune modulatory function(s) of Sema-3E on natural killer (NK) cells.

## 1. The Semaphorin Family: Classification and Structure

**Figure 1:**
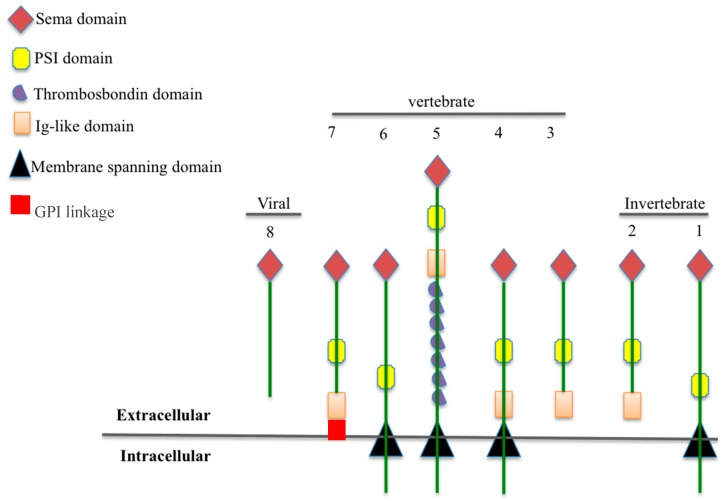
Classes and structures of semaphorins. Semaphorins are represented in their classification into eighth classes. Class 1 and 2 semaphorins are found in invertebrates. Class 3–7 semaphorins are found in vertebrates. The Sema domains characterize both semaphorins and plexins. Additional domains present in semaphorins and plexins include PSI domains (plexin, semaphorin, and integrin) and immunoglobulin (Ig)-like domains. Class 7 semaphorin (Sema-7) contains a membrane-associated GPI moiety at its carboxyl terminus. Class V (Sema-8) semaphorins are highly similar to the Class 7 semaphorins and are found in DNA viruses. Semaphorins can be either secreted or membrane-bound proteins. Note: Some members of the Class 4 semaphorins can be found in secreted form. Some members of the Class 3 proteins can be found on cell surfaces.

Semaphorins were first discovered as axon guidance molecules in the nervous system [1,2]. Currently, they represent a large family of proteins that are classified into eight classes (1–7 and V). Classes 1 and 2 are found in invertebrates. Classes 3–7 exist in vertebrates, whereas Class V is unique to viruses [3]. The differences between these classes are related to their sequences and structures. A signature domain, however, called the semaphorin (Sema) domain, is conserved among all members of the semaphorins family. The extracellular Sema domain consists of 500 amino acids and is a cysteine-rich sequence that is crucial for receptor binding specificity and protein functions (Figure 1) [4,5,6,7]. Semaphorin molecules can be membrane-bound (Classes 1, 3, 4, 5, and 6) [8,9], secreted proteins (Classes 2, 3, 4, and V) [10,11], or glycosyl–phosphatidyl–inositol (GPI)-linked proteins (Class 7) [8].

The plexin receptors are large 200 kDa transmembrane proteins that have been identified in vertebrates (Plexins A1–A4, B1–B3, C1, and D1) and two in invertebrates (Plexins A and B) [3]. The extracellular part of the plexin receptors contains a Sema domain, followed by three PSI (plexin–semaphorin–integrin) domains and three IPT (immunoglobulin, plexin, and transcription factors) domains [12]. The PSI domain is a small cysteine-rich domain that is crucial for protein–protein interactions [13]. The IPT domain is required for proper ligand binding to the plexin receptors. The intracellular domain or cytoplasmic tail of the Plexin molecule is highly conserved and plays a crucial role in transmitting the signals following ligand binding. It contains a putative tyrosine phosphorylation sites, a GTPase-binding domain, and a segmented GTPase-activating protein (GAP) domain [14,15,16]. Neuropilins (NRP) receptors, NRP1 and NRP2, are single-pass transmembrane proteins that contain short cytoplasmic tails. The extracellular portion of NRPs contains two repeat complement-binding (CUB) domains (a1 and a2 domains), two coagulation factor-like domains (b1 and b2 domains), and a juxta-membrane meprin/A5/mu-phosphatase (MAM) homology domain (c domain) [17].

Selective binding and signaling of individual Semaphorin members is thought to be determined by the receptor complexes that can exist either as homomeric or heteromeric complexes [5]. Most semaphorin molecules mediate their effector functions by direct binding and signaling of plexins and neuropilins (NRPs) receptors [15]. For example, Sema-4A has 4 types of receptors: the Plexin-D family, the Plexin-B family, Tim-2 (T-cell, immunoglobulin, and mucin domain protein 2), and NRP-1. In most cases, members of the Plexin-A family require neuropilins as ligand-binding partners, whereas members of the other plexin families are directly activated by semaphorins [18]. Binding of Semaphorin-3 family members to neuropilin (NRP) receptors depends on their N-terminus Sema sequences, and whether the 70-amino acid stretch within these sequences will determine binding specificity [6,19]. NRP receptors may lack intrinsic signaling capabilities due to their short cytoplasmic tails [20]; however, both NRP1 and NRP2 were found to be essential for semaphorin-3-induced signal transduction [21,22]. Blocking of NRP1 (receptor of Sema-3a) using anti-NRP1 antibodies resulted in ablation of the axon repulsion of mouse cortical neurons [23]. Knock-out of the NRP-1 gene expression caused semaphorin-3a insensitivity in embryonic DRG neurons [24]. NRP-2 is an essential component for semaphorin-3f function. Blocking of the NRP-2 receptor abolished the semaphorin-3f-induced growth cone collapse of embryonic rat sympathetic neurons, and axon repulsion in neonatal mouse cortical neurons [21]. The development of dopaminergic neurons in the meso-diencephalon was impaired in mice that were deficient in NRP2 [25]. It is therefore unclear what signal transducers are involved in the downstream signaling of the NRP receptors. Depending on the cell type, other membrane-associated proteins such as vascular endothelial growth factor receptor (VEGF) or CD72 could act as co-receptors for specific semaphorin members to mediate its effector functions [13]. For examples, Class 6 semaphorins bind to Class A plexin receptors and carry out different biological activities depending on its VEGF co-receptor [13].

In the neuronal system, semaphorins can mediate repulsive axon guidance, cell migration, invasive growth, and growth cone collapse by several post-translational modifications [26] and oligomerization [27]. The importance of semaphorins in regulating cellular events beyond the nervous system is emerging. Members of the semaphorin family are reported to play important roles in immune, respiratory, and cardiovascular systems, in physiological processes such as angiogenesis, embryogenesis, and in pathological conditions such as airway diseases and tumor formation [28,29,30,31,32,33]. This review focuses on the recent advances in our understanding of the semaphorin-3E (Sema-3E) member of the family in these processes, and its emerging roles in regulating immune responses.

## 2. Receptors and Signaling of the Semaphorin-3E

**Figure 2:**
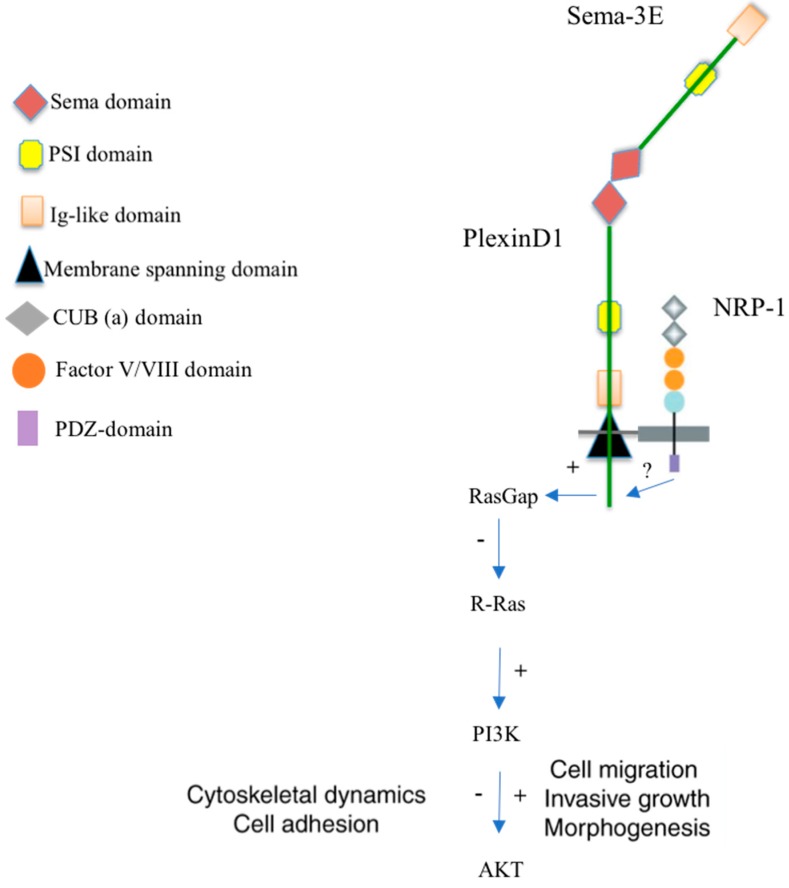
The Sema-3E/Plexin-D1 receptor signaling. Sema3E binds its receptor, Plexin-D1. This interaction leads to the activation of the intracellular Plexin-D1 RasGAP (Ras GTPase activating protein) domain and subsequently reduces R-Ras activity [37]. Semaphorin-3E can regulate integrin functions and cytoskeletal dynamics via the intrinsic R-Ras GAP activity of plexins and the recruitment of regulatory molecules, and can thereby affect cell adhesion and migration. These effects can sometimes result in opposing functional responses, depending on the activation/inhibition of PI3K (phosphoinositide 3-kinase), in a cell-type-specific manner. The role of NRP-1 remains unclear. It could function to inhibit repulsive signaling by Plexin-D1, thereby facilitating attractive/growth-promoting responses. (+) Activation; (−) Inhibition; (?) Unknown.

The Sema-3E gene is located on Chromosome 7 and encodes a 85–90 kDa protein. Unlike the other Semaphorin-3 members, Sema-3E can bind to the Plexin-D1 receptor with high affinity, independent of the NRP [4,33,34,35]. Intracellular tail of Plexin-D1 contains two highly conserved intracellular domains—the SEX-PLEXIN domain and the SEMA/PLEXIN domain. The SEMA/PLEXIN domain of Plexin-D1 includes two C region RasGAP domains. Each RasGAP domain includes a short motif of (GTPase)-activating proteins (GAPs) and monomeric GTPases of the R-Ras subfamily (Figure 2). A monomeric Rho GTPase binding domain (RBD) is found within the two C regions. Plexin-D1 acts as a RasGAP to antagonize both an integrin-mediated cell extracellular matrix (ECM) adhesion and PI3K, a modulator of cell survival, growth, and migration signaling. Rho family GTPase 2 (Rnd2) is required for the activation of the RasGAP activity of Plexin-D1. Upon Sema-3E Plexin-D1 stimulation, pre-existing Plexin-D1-Rnd2/RLG (resistance-nodulation division/Release Guard signal) complexes undergo Rnd2/RLG-dependent intracellular conformational changes that translate the concentration and distribution of extracellular Sema-3 cues into an intracellular gradient of distinct Plexin-D1 activities [20,36]. Multifaceted functions of semaphorins may also be mediated by other signaling pathways, such as mitogen-activated protein kinase or phosphatidylinositol 3-kinases. The mechanism underlying how the binding of semaphorins induces a network of downstream signaling has not been fully deciphered.

## 3. Semaphorin-3E in the Nervous System

Sema-3E proteins are present in the neural scar and influence a wide range of molecules and cell types in and surrounding the injured tissue [38]. The Sema-3E–Plexin-D1 axis has a dual function in axonal growth depending on the presence of NRP1. For certain subpopulations of corticofugal and striatonigral neurons that express Plexin-D1 but not NRP1, Sema-3E acts as a repellent. In contrast, in subiculo-mammillary neurons, the presence of receptor complexes of NRP1 in addition to Plexin-D1 switches the Sema-3E signal from repulsion to attraction and/or stimulation of axonal growth [39]. Recently, Sema-3E–Plexin-D1 signaling is involved in synaptic recognition in the spinal cord and striatum. In the spinal cord, Sema-3E–Plexin-D1 plays a role in the specificity of monosynaptic sensory-motor connections [40]. Therefore, in the spinal cord post-synaptic neurons releasing guidance cue, Sema-3E, and repel incoming axons that express Plexin-D1 to prevent inappropriate synapse formation [41]. Ding et al. reported that Sema-3E was secreted by incoming thalamic axons and that Plexin-D1 expressed by one subtype of post-synaptic neuron could specify synaptic specificity [42]. Collectively, Sema-3E–Plexin-D1 signaling determines synaptic recognition and specificity in multiple parts of the nervous system [34].

## 4. Semaphorin-3E in Cardiovascular Development

Sema-3E was discovered to play crucial roles in cardiovascular development, mainly acting through NRP1 and Plexin-D1 [43]. Many studies have been focused on Sema-3E signaling in vascular patterning and cardiac morphogenesis, and Sema-3E signaling impairment has been associated with various human cardiovascular disorders, such as persistent truncus arteriosus, sinus bradycardia, and anomalous pulmonary venous connections [44]. Sema-3E–Plexin-D1 signaling is required for proper dorsal aortae patterning in the early embryo [45]. This signaling can repress angiogenesis by antagonizing the proangiogenic activity of VEGF [46]. Moreover, Sema-3E affects retinal angiogenic cell fate decisions by regulating cell responsiveness to VEGF and Notch in tip and stalk cells [47].

## 5. Semaphorin-3E and Cancers

High levels of expression of Sema-3E and Plexin-D1 were observed in human colon cancer, liver metastasis, and melanoma progression [17,48]. Further functional analyses revealed that Sema-3E could regulate invasiveness of tumor cells in a Plexin-D1-dependent manner [49,50]. In breast cancers, expressions of Sema-3E and the Plexin-D1 receptor have been shown to be upregulated in advanced and metastatic human breast tumors. The Sema-3E/Plexin-D1 signaling promoted survival of breast cancer cells. Suppression of such Sema-3E/Plexin-D1 signaling pathway in human and mouse breast cancer cells induced apoptosis in vitro and subsequently reduced metastasis in vivo [48,49,51].

## 6. Semaphorin-3E in an Allergic Asthma Model

Emerging data suggest that semaphorins and their receptors are key regulators of allergic inflammatory responses in the airways [32]. Of particular interest to us, we observed that expression of Sema-3E was significantly suppressed in the airways of severe asthmatic patients [52] and in an experimental mouse model of asthma [53]. In addition, the surface expression of the Plexin-D1 receptor was reduced in the airway smooth muscle cells from asthmatic patients, thus suggesting the functional importance of Sema-3E/Plexin-D1 signaling in allergic asthma [54]. We reported that Sema-3E inhibited human airway smooth muscle (ASM) cell migration and proliferation by modulation of Rac1, ERK1/2, and Akt pathways [54]. To further examine the role of Sema-3E in the development and maintenance of allergic asthma, we used Sema-3E-deficient mice in experimental models of asthma. We observed that genetic ablation of Sema-3E in mice resulted in increased lung granulocytosis, increased airway hyper-responsiveness, mucus overproduction, collagen deposition, and Th2/Th17 lung inflammation in allergic asthma [53]. The regulatory role of Sema-3E in allergic asthma seems to be mediated by the modulations and/recruitment of pulmonary dendritic cell (DC) subset [53] and neutrophils [55]. Intranasal administration of recombinant Sema-3E alleviated these pathological features of experimental allergic asthma, highlighting the importance of Sema-3E in maintaining a homeostatic balance in the airway [56].

## 7. The Prospective Role of Semaphorin-3E in Regulating Natural Killer (NK) Cell Functions

NK cells are bone-marrow-derived cells that constitute 10–15% of blood lymphocytes [57]. They migrate to peripheral tissues or inflamed lymph nodes to exert their immune-surveillance functions [58]. They are currently classified as members of the emerging family of the innate lymphoid cells that play important roles in innate immunity and tissue remodeling [59,60]. NK-cell activation and function can be regulated by target cell recognitions, cytokines (such as IL-2, IL-12, IL-15, IL-18) [61], or dendritic cells (DCs) in microenvironments [62,63]. The interaction of NK and DCs (crosstalk) is bi-directional, involving multiple cytokine signals and direct cell–cell contacts [64,65,66,67]. DC-derived cytokine IL-12 is critical in the generation of IFN-γ-producing NK cells. Interestingly, DCs also derive soluble factors such as IL-1 and IL-18, which have implications in terms of the acquisition of IL-12 receptor on NK cells [68]. Mutually, NK cells promote DC maturation and activation by inducing MHC molecule expression and by enhancing the ability to secrete IL-18, IL-12, and P70 via upregulation of CD86 molecules and the activation of Triggering Receptor Expressed on Myeloid Cells 2 (TREM2) and NKp30 signaling [69,70]. As DCs can acquire different abilities to induce immunological tolerance or to stimulate functionally distinct T cell subsets (such as Th1, Th2, and Th17) effectively [71], the regulation of DC maturation/functions by NK cells is important in coordinating innate and adaptive immune responses. NK–DC crosstalk therefore shapes anti-tumor and anti-microbial responses in vivo [64,67,72,73,74,75,76].

Holl et al. reported that Plexin-B2 and Plexin-D1 are reciprocally expressed on plasmacytoid and myeloid DCs. The predominant expression of the Plexin-D1 receptor on bone-marrow-derived DCs (BMDC) can be further modulated by TLR ligands [77]. In addition, splenic DCs expressed high levels of Sema-3E [77]. Plexin-D1-deficient and wild-type DCs exhibited comparable LPS-induced DC maturation, T-cell stimulations, and migrations towards CXCL12/19 gradients. However, the Plexin-D1-deficient DCs were hyper-responsive in their secretion of IL-12/IL-23 p40 (but not IL-6) when these sorted splenic DCs were cultured in vitro at the steady state for 24 h [77]. It will be interesting to examine how modulation of IL-12 production by Plexin-D1/Sema-3E may further regulate NK cell activation, NK cell functions, or NK–DC crosstalk in vitro and in vivo.

Our recent work demonstrated that mouse NK cells expressed Sema-3E receptor (Plexin-D1) on their cell surface [78]. It thus highlighted the possibilities of a direct regulatory effect of Sema-3E on NK-cell functions. We observed also the expression of Sema-3E in bone-marrow-derived DCs was tightly regulated in DC maturation [78]. It will be interesting to examine further, for example, how Sema-3E production by DCs regulates NK cell functions, NK-induced DC maturation, or DC homeostasis in NK–DC crosstalk.

## 8. Conclusions

Semaphorins were first identified as axon guidance cues in neural development. Recent work has established its multi-faceted role in the cardiovascular system, airway biology, cancers, and immune cell regulation. Future investigations of the role(s) of Sema-3E in regulating NK cells and/or NK–DC crosstalk will provide new insights into the importance of Sema-3E in maintaining the homeostasis of immune cells in physiological and pathological settings.
